# Incidentally Detected Odontogenic Keratocyst With Inconclusive Histopathology: A Case Report Emphasizing Clinicoradiologic Correlation

**DOI:** 10.1155/crid/6449344

**Published:** 2026-04-30

**Authors:** Ghaida AlJamal, Wlla E. Al-Hammad, Saleh N. Almohammed, Rima A. Safadi, Abdalla Hazza’a, Taiseer H. Al-Khateeb

**Affiliations:** ^1^ Department of Oral Medicine and Oral Surgery, Faculty of Dentistry, Jordan University of Science and Technology, Irbid, 22110, Jordan, just.edu.jo; ^2^ Department of Prosthodontics, Faculty of Dentistry, Jordan University of Science and Technology, Irbid, 22110, Jordan, just.edu.jo

**Keywords:** cone beam computed tomography, jaw cysts, multidisciplinary diagnosis, odontogenic keratocyst

## Abstract

Odontogenic keratocysts (OKCs) are locally aggressive developmental odontogenic cysts that may complicate diagnosis and management. A 61‐year‐old male underwent CBCT for implant planning. The scan showed a pericoronal lesion with a scalloped margin and minimal expansion suggestive of OKC. The lesion was enucleated; however, histopathology was inconclusive because of inflammation and recommended clinicoradiologic correlation. Follow‐up was advised because of the lesion’s recurrence potential. This case highlights the need for a systematic review of the full CBCT volume and multidisciplinary correlation when classic histologic features are obscured by inflammation.

## 1. Introduction

Odontogenic keratocysts (OKCs) are not common, with a reported prevalence of around 10%–12% depending on the population [1]. They are benign developmental lesions that may behave locally aggressively. The classification of OKC has been revised several times by the World Health Organization because of debate about tumor‐like behavior and histopathological characteristics [2]. These cysts most often arise in the posterior mandible, particularly the ramus‐angle region; they tend to remain clinically silent until imaging and have a high recurrence rate. Patients with infected lesions may present with fever, discharge, or tooth mobility [3, 4].

OKCs uncommonly present as a unilocular or multilocular radiolucent area with minimal buccolingual expansion and a tendency to grow anteroposteriorly within the medullary bone. Three‐dimensional imaging techniques enable evaluation of the lesion margins, its internal structure, and its effects on adjacent anatomical structures. Consequently, CBCT plays a crucial role in supporting clinicoradiologic correlation [5]. OKCs are typically asymptomatic and are often discovered incidentally during routine radiographic evaluation for unrelated dental issues [6, 7]. Despite imaging advantages, some OKCs may remain undetected. There is a risk of overlooking this lesion on CBCT when it is reviewed narrowly for a specific treatment objective, such as implant planning. Misdiagnosis of OKCs is not uncommon because they resemble other lesions, such as dentigerous cysts. The association with embedded or impacted teeth further complicates the diagnosis [8]. They also mimic ameloblastoma, especially when large or multilocular, but OKC rarely causes root resorption or any bony expansion, which is common in ameloblastoma [3, 9].

Histopathological examination remains the gold standard for diagnosis of OKCs and determining the most effective management to prevent recurrence [10]. OKC typically shows uniform parakeratinized stratified squamous epithelium; however, in some cases, the lining is discontinuous and inflamed, especially when cysts are secondarily infected. These changes may reflect previous cyst rupture, chronic inflammation, or sampling from nonrepresentative areas of the lesion, which can mask the classical features and complicate the diagnosis [11].

In such cases, radiographic characteristics become an important complementary aid to inform diagnostic reasoning [12]. Oral maxillofacial radiology is critical to identify such lesions on CBCT and prevent delays in diagnosis and treatment, even when the scans are acquired for other purposes.

Multidisciplinary collaboration between radiologists, surgeons, prosthodontists, and histopathologists is essential to reach the proper diagnosis and develop the optimal treatment plan. In this case report, radiographic features were highly suggestive of OKC and, together with the pathologist’s recommendation for clinicoradioglic correlation, supported the working diagnosis. This case highlights how radiographic features support diagnosis through correlation. It also underlines the clinical importance of comprehensive CBCT interpretation to avoid overlooking clinically relevant findings.

## 2. Case Presentation

**Figure 1:**
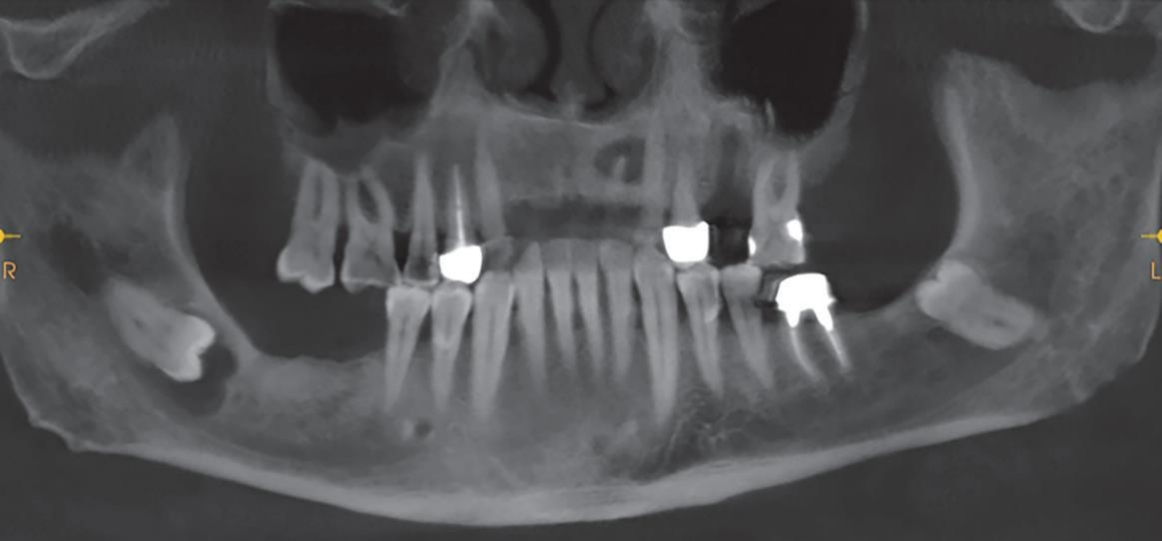
Reformatted panoramic image showing an impacted right mandibular third molar.

**Figure 2:**
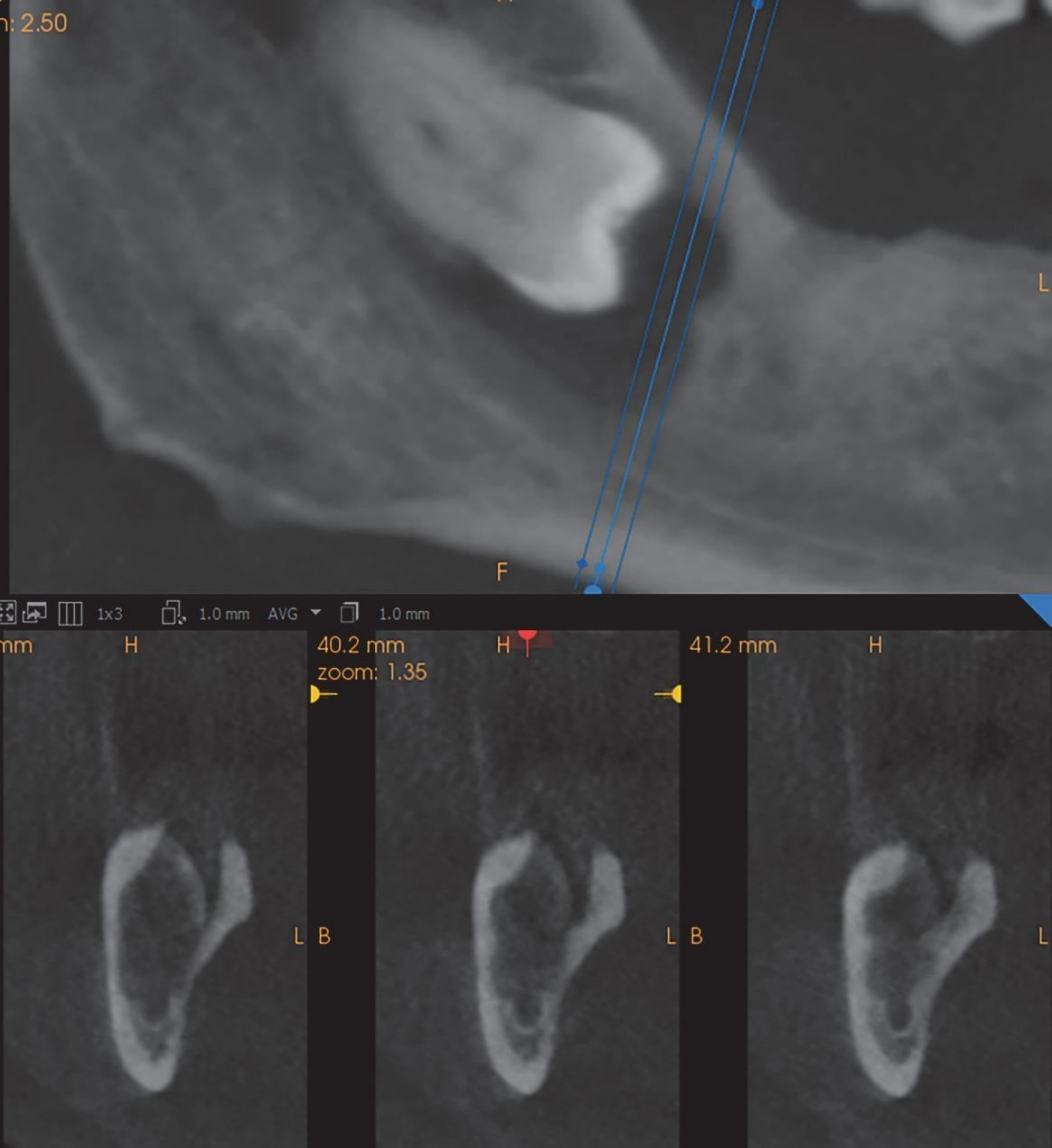
(Top) Cropped, reformatted panoramic image showing a pericoronal radiolucency associated with the impacted right mandibular third molar. (Bottom) Buccolingual CBCT cross‐sections demonstrate a well‐defined lesion with scalloped borders and no cortical expansion.

A 61‐year‐old hypertensive male smoker visited the prosthodontic clinic at the Dental Teaching Clinics, Faculty of Dentistry, Jordan University of Science and Technology, in November 2024, seeking replacement of multiple missing teeth. Following clinical examination, the prosthodontist referred the patient for CBCT to evaluate the potential sites for implants. CBCT images revealed impacted mandibular third molars that were close to the inferior alveolar canal (Figure 1). Moreover, the right third molar showed a pericoronal multilocular radiolucent lesion that was mostly well‐defined, except at its superior extent near the external oblique ridge and at its inferior border (Figure 2, top). The lesion was corticated except for the previously mentioned ill‐defined areas. Buccolingual cross‐sectional images showed a scalloped border with no expansion of the buccal or lingual cortical plates (Figure 2, bottom). Intraoral and extraoral examination revealed no clinical signs of swelling or abnormality. The patient reported that he had multiple episodes of discharge from the most posterior mandibular region. He had not sought dental care or taken any medication at that time. The lesion in the right third molar was enucleated under local anesthesia and was submitted for histopathological examination. During enucleation, the oral and maxillofacial surgeon who favored a dentigerous cyst in the differential diagnosis was concerned about the lesion’s nature and suspected OKC based on intraoperative appearance. Therefore, after enucleation of the cyst and tooth extraction, peripheral ostectomy at the bony bed was done to an approximate depth of 3 mm in all directions with caution over the inferior alveolar neurovascular bundle.

**Figure 3:**
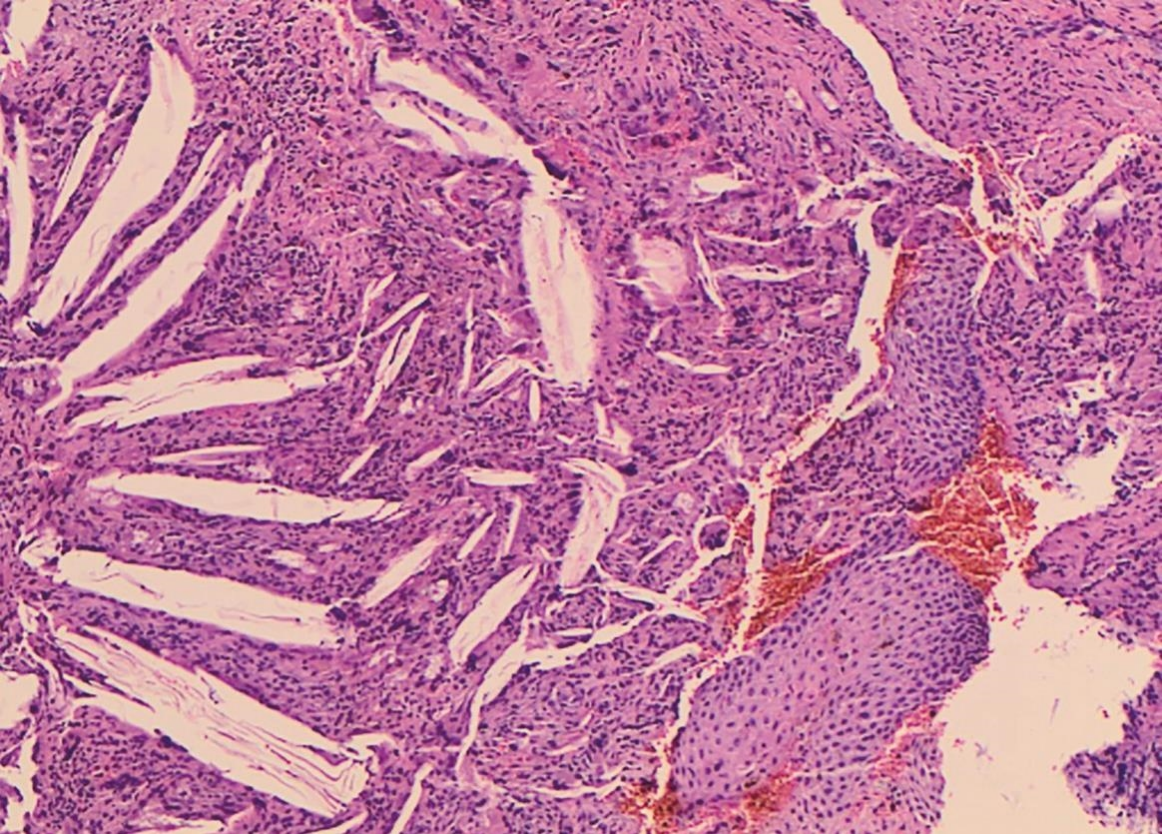
Hematoxylin and eosin‐stained section, showing intense inflammatory infiltrate in the cyst wall with multiple cholesterol clefts formation. The lining epithelium is reactive hyperplastic with no specific features. Image taken at 10x.

**Figure 4:**
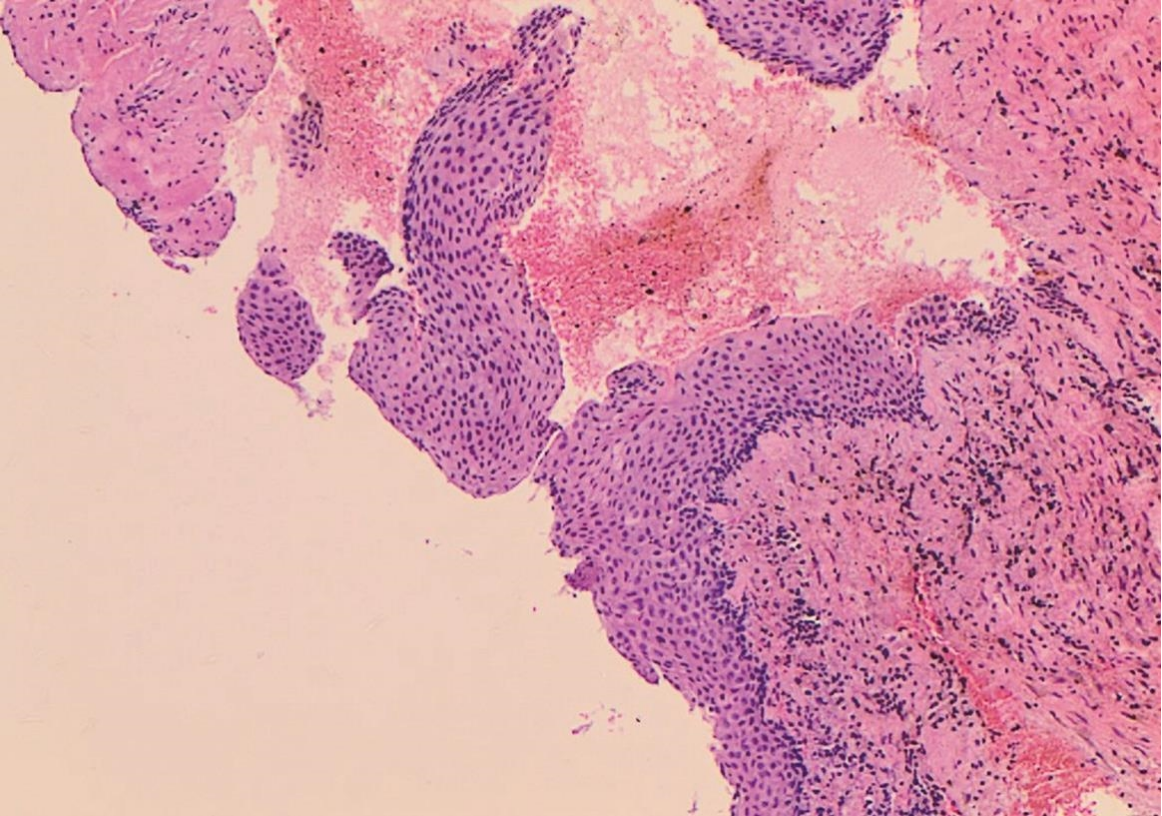
Hematoxylin and eosin‐stained section, showing chronic inflammation in the cyst wall. The lining epithelium shows some but inconclusive features of odontogenic keratocyst. Image taken at 10x.

The histopathologic examination revealed an extensively inflamed cyst with hyperplastic squamous epithelial lining, foreign material, and foreign body reaction (Figure 3). The typical histopathologic features of OKC were altered to a nonspecific cyst lining. However, a localized full‐thickness sloughing of a focus of the cyst lining epithelium was noted in addition to other focal areas of lining epithelial surface corrugation (Figure 4). Tendency for uniform lining epithelial thickness was seen in other localized areas, leading to the comment that OKC cannot be excluded and clinicoradiologic correlation was recommended (Figure 4).

**Figure 5:**
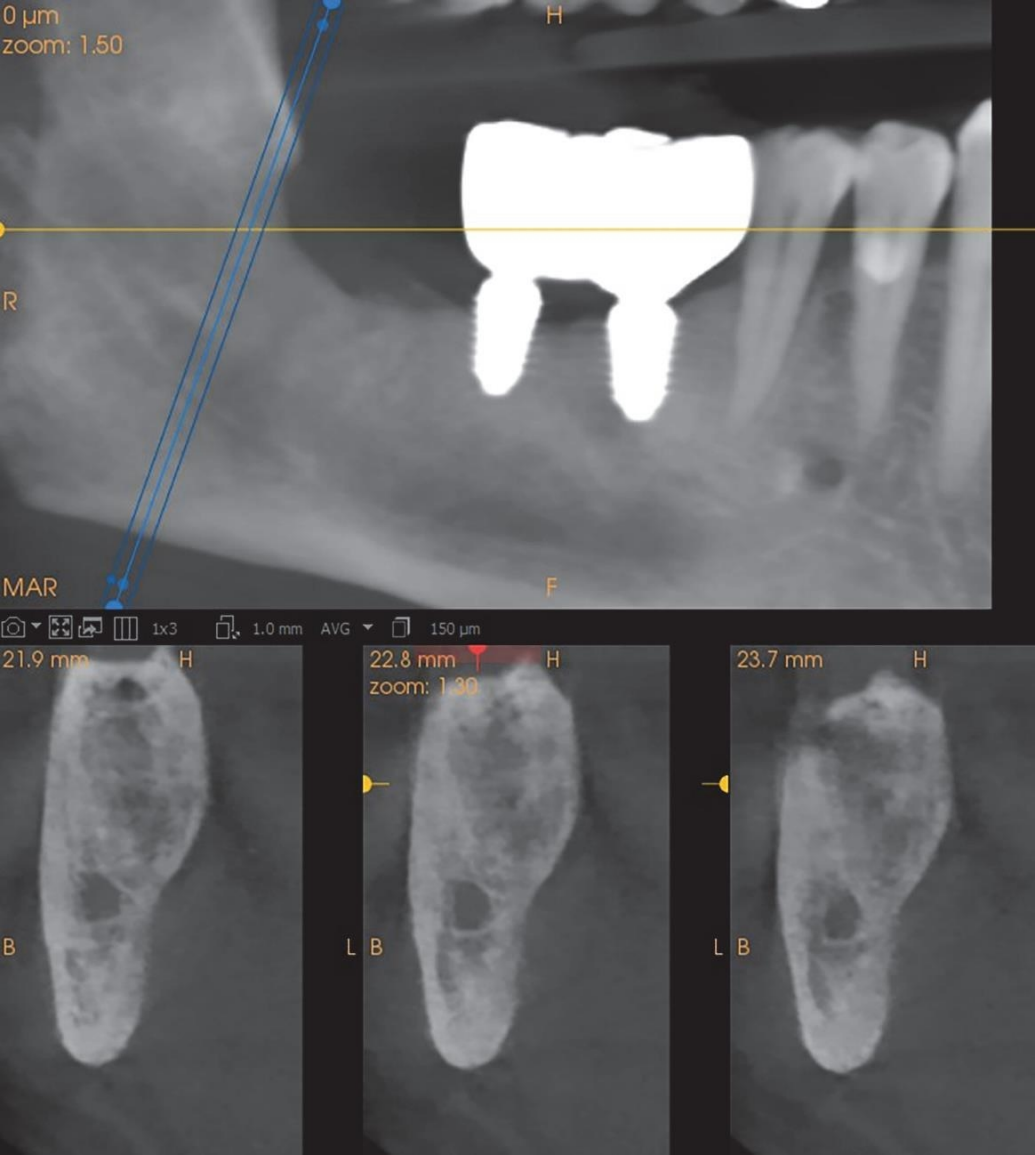
(Top) Cropped reformatted panoramic image of the right mandibular region showing healing in the area of the extracted right mandibular third molar. (Bottom) Buccolingual cross‐sectional CBCT images demonstrate healing and bone formation in the area with intact cortical borders and no sign of recurrence.

The patient was followed up both clinically and radiographically at 6‐month intervals, and so far, no recurrence has been detected at 12 months (Figure 5).

## 3. Discussion

While OKCs are diagnosed in younger individuals, they may be encountered in older adults and are sometimes detected on implant‐planning CBCT, with a higher prevalence noted in males [13]. The management of these lesions in older individuals may be more complex, as they have comorbidities that may influence treatment planning and follow‐up [2]. Given the recurrence potential of OKCs, regular follow‐up is recommended.

On CBCT, OKCs are often seen as well‐defined, radiolucent lesions exhibiting smooth or scalloped borders with minimal expansion. These cysts are found most commonly in the posterior mandible (ramus/angle) and can occur in the maxilla [4]. Three‐dimensional imaging offers clinicians clearer visualization compared to conventional two‐dimensional radiographs; it delineates the extent and relationship to the inferior alveolar canal and cortical plates [14]. This supports clinicoradiologic correlation and presurgical assessment in anatomically complex regions like the third molar area.

In cases of infected cysts, CBCT may show less typical margins and adjacent inflammatory changes. In the present case, the lesion lining was inflammation‐altered, and on CBCT, it showed focal irregular areas with poorly defined borders. In some cases, the surrounding bone shows features of osteomyelitis, including bone resorption, irregularity, and loss of cortical continuity, which contrasts with the smooth corticated border seen in noninfected OKCs [15]. CBCT may show these changes, evaluate the extent of the infection, and assist in differentiating between a simple OKC and one that has developed an infectious complication. This can guide surgical planning, including the need for drainage, antibiotics, or more aggressive surgical debridement [16]. Although histopathological examination is the gold standard for diagnosing OKCs, in this case, the biopsy contained an extensively inflamed cyst wall with cholesterol clefts, giant cells, mixed inflammatory infiltrate. This resulted in hyperplastic lining epithelium with elongated rete ridges instead of the characteristic OKC epithelial lining of ribbon‐shaped, 5–8 cell thick with corrugated surface and clean separation from underlying cyst wall [15]. A minor focus of a small fragment of epithelial lining retained some features of OKC, leading to a comment in the histopathology report that OKC cannot be completely excluded and that clinicoradiological correlation is recommended.

In this case, the lesion was incidentally detected on implant‐planning CBCT in an asymptomatic patient. A dentigerous cyst was considered because it is the most common pericoronal radiolucency in the jaws affecting the mandibular third molar. Imaging features favored OKC as the lesion was multilocular with scalloped margins and caused no significant expansion or displacement of affected teeth [16]. A dentigerous cyst is typically attached at the CEJ radiographically, so it was less favored as the lesion extended beyond the CEJ. Central mucoepidermoid carcinoma, although rare, was considered because it sometimes presents as a multilocular radiolucency, often with well‐defined or irregular borders and given the patient’s age and the focal ill‐defined margin [17, 18].

## 4. Conclusion

This case report emphasizes clinicoradiologic‐pathologic correlation in the diagnosis and management of OKCs, particularly when histopathological findings are inconclusive. While histopathology is an important diagnostic tool, advanced imaging techniques supported diagnostic interpretation and treatment planning. Early detection through radiological assessment, combined with histopathology that recommended clinicoradioglogic correlation, is important for management and reducing the risk of recurrence. Imaging features and a multidisciplinary approach support comprehensive patient care, from initial imaging to surgical planning, supporting management decisions for patients with locally aggressive lesions like OKCs. At the most recent follow‐up, the patient was clinically stable, and follow‐up CBCT showed radiographic evidence of healing at the surgical site.

## Author Contributions

**Ghaida AlJamal:** conceptualization, writing – original draft, writing – review and editing, project administration. **Wlla E. Al-Hammad:** methodology (radiologic assessment), resources, writing – review and editing. **Saleh N. Almohammed:** investigation, data curation, writing – review and editing. **Rima A. Safadi:** methodology (histopathologic assessment), validation, formal analysis. **Abdalla Hazza’a:** supervision, validation, writing – review and editing. **Taiseer H. Al-Khateeb:** conceptualization, supervision, writing – review and editing.

## Funding

This research received no external funding.

## Disclosure

All authors read and approved the final manuscript.

## Ethics Statement

This report was prepared in accordance with the Declaration of Helsinki. Written informed consent was obtained, authorizing the publication of clinical details and images, including CBCT scans and histopathology photographs. All images were deidentified and no identifying patient information is included.

## Conflicts of Interest

The authors declare no conflicts of interest.

## Data Availability

The data that support the findings of this study are available from the corresponding author upon reasonable request.
